# Feasibility of digital healthcare in enhancing healthcare access in semiurban areas of Karachi, Pakistan: a qualitative descriptive study

**DOI:** 10.1136/bmjopen-2023-082558

**Published:** 2025-07-11

**Authors:** Narjis Rizvi, Romaina Iqbal, Rawshan Jabeen, Bronwyn Harris, Frances Griffiths

**Affiliations:** 1 Community Health Sciences, The Aga Khan University, Karachi, Pakistan; 2 Division of Health Sciences, Warwick Medical School, University of Warwick, Coventry, UK; 3 Centre for Health Policy, School of Public Health, University of the Witwatersrand, Johannesburg, South Africa; 4 Warwick Medical School, University of Warwick, Coventry, UK

**Keywords:** Telemedicine, Health informatics, Health Services Accessibility

## Abstract

**Abstract:**

**Objective:**

Our research aimed to assess the feasibility of digital health in enhancing healthcare access in the semiurban areas of Karachi, Pakistan.

**Study design and setting:**

This qualitative descriptive study was employed at three villages in Gadap, Karachi, Pakistan, with varying socioeconomic contexts, using a feasibility framework. Ethical approval was provided by the Ethical Review Committee (ERC) of The Aga Khan University.

**Study participants:**

Through purposive sampling, demand and supply-side stakeholders (N=152) were invited to participate in the study, including community leaders, activists and members, representatives from non-governmental organisations, public and private sector healthcare providers, and digital healthcare providers and experts. Both inductive and deductive approaches were used for data analysis.

**Measure of outcomes:**

The assessment of feasible demand-side and supply-side factors would be extremely useful in the planning and implementation of a sustainable digital health programme.

**Results:**

Digital health is an acceptable and practically feasible option and is a potential solution to enhance healthcare access and equity, particularly in semiurban and rural remote areas, where healthcare access is limited. Digital healthcare should not replace inperson healthcare but should instead be offered in combination with it, preferably through a ‘Hub & Spoke Model’ facility*.* Few challenges exist in implementing digital health, including privacy, ethical issues, lack of evidence-based standards, inadequate training of healthcare providers, technological barriers and access to digital health services by vulnerable populations, such as the elderly, women, individuals who are illiterate and those of low-income class.

**Conclusion:**

Our study concludes that digital healthcare is a dire need and is a potential solution to enhance healthcare access and equity, as it is acceptable and practically feasible. A mix of inperson and digital health consultation should be offered through a hub and spoke model. Few challenges to implementing digital health exist and should be addressed by tailoring digital health through co-creation and engaging all stakeholders.

**STRENGTHS AND LIMITATIONS OF THIS STUDY**

- Our study provides a comprehensive understanding of the feasibility of digital healthcare delivery systems in the semiurban and rural areas of Karachi, Pakistan.
- The study used an objective framework to gather the perspectives of demand-side and supply-side stakeholders on the key domains of feasibility of digital healthcare delivery.
- The study purposively selected and engaged all relevant stakeholders for a comprehensive understanding of the planning steps and the processes required to introduce digital healthcare to the existing healthcare delivery system.
- Because of the unique features of the semiurban areas of Karachi, the results might not be applicable to other areas of the country, highlighting the need for further research in other areas of Pakistan.

## Introduction

 Universal health coverage (UHC) is a global goal.^1^ The UHC service coverage index (Sustainable Development Goal (SDG) indicator 3.8.1) has increased from 45 in 2000 to 67 in 2019; however, 30% of the world’s population still cannot access essential health services.^2^ Achieving UHC is more challenging in low-income and middle-income countries (LMICs),^3^ where the public health system is fragile and has inadequate resources to cater to the health needs of the population. Furthermore, the health needs in LMICs are increasing due to the ‘Triple Disease Burden’ and the rising trend of pandemics.^4 5^ For example, the COVID-19 pandemic has devastated the global economy^6^ in general, but has an enormous impact in LMICs.^7^ Policy adaptations resulting from wrecked economies have further slowed the progress in achieving UHC in LMICs.^8^

UHC aims to enhance access to the full continuum of essential health services.^2^ Studies show that lack of access to healthcare is a universal phenomenon; however, vulnerable populations in LMICs are affected the most.^9^ Governments around the world are facing the challenge of providing equitable healthcare services to all their citizens.^10^ In general, residents of rural and remote areas have less access to healthcare compared with people living in larger cities.^11^,^14^

Innovative models for healthcare delivery have been conceived and piloted to enhance access to healthcare and hence improve health indicators.^15^ One of these innovations is ‘Digital Health’, ‘a broad umbrella term encompassing eHealth (which includes telemedicine and mHealth), as well as emerging areas, such as the use of advanced computing sciences in ‘big data’, genomics and artificial intelligence’.^16^ All WHO member states passed a resolution in May 2018 to encourage the recognition of the value of digital health in contributing to advancing UHC and other health aims of the SDGs. All WHO member states also passed a resolution in May 2018^17^ encouraging the ministries of health ‘to assess their use of digital technologies for health and to prioritize, as appropriate, the development, evaluation, implementation, scale-up and greater use of digital technologies’.^18^ Studies show that telemedicine is one of the innovations that have enhanced access to healthcare and medical knowledge,^19 20^ with reduced need for travel.^21^ ‘Telemedicine’, which literally means ‘healing at a distance’, is defined broadly as ‘the use of information communication technology (ICT) to deliver medical services at a distance’.^22^ Telemedicine was first used in the 1970s to refer to the application of ICT to improve patient outcomes by enhancing access to care and medical knowledge.^23^ Various terminologies used for telemedicine include electronic health (eHealth) and mobile health (mHealth) (refer to online supplemental annexure 1).

Realising digital health as a potential solution to enhance access to healthcare, many digital health initiatives have been launched. Some of these digital health platforms have been scaled, but most could only reach prototype development/pilot testing and could not be implemented due to limited funding, absence of a business model, lack of stakeholder ownership in public and private sectors, technological savviness and absence of legislation.^24^ Even projects that were implemented, either their uptake in the mainstream health system has been much slower than the expected timeline or they were not sustainable.^25 26^

Based on internet-based mapping of the existing digital health in Karachi, Pakistan, two categories of digital health services were identified, namely ‘Digital Healthcare Platforms’ and ‘Individual Digital Healthcare Services’. This mapping revealed that seven digital healthcare platforms exist in Karachi, Pakistan; five deliver consultation services for general health issues, two provide maternal health services and prescribe medicines, two offer referrals, and one offers all health services for women through an app and video/phone consultations. Consultation fees range widely across these digital healthcare platforms, from US$2 to US$30; one provides free services. In the category of ‘Individual Digital Healthcare Services’, community members approach local health workers using their own phones.

Evidence shows that various demand-side and supply-side factors affect the utilisation of healthcare services.^27^ Recognising the vital role that demand-side and supply-side factors play in healthcare utilisation, we designed this study to assess the feasibility of digital health in enhancing healthcare access from the demand-side and supply-side perspectives in terms of acceptability, practicality, adaptation and integration into existing health services. The demand-side factors are defined as user characteristics, while the supply-side factors are healthcare system characteristics.^28^ The assessment of the feasibility of the demand-side and supply-side factors would be extremely useful in the planning and implementation of a sustainable digital healthcare programme. This study engaged relevant demand-side and supply-side stakeholders to assess the context and feasibility of digital healthcare in enhancing the healthcare access of semiurban and rural residents of Karachi, Pakistan.

## Methods

### Study design and purpose

**Figure 1:**
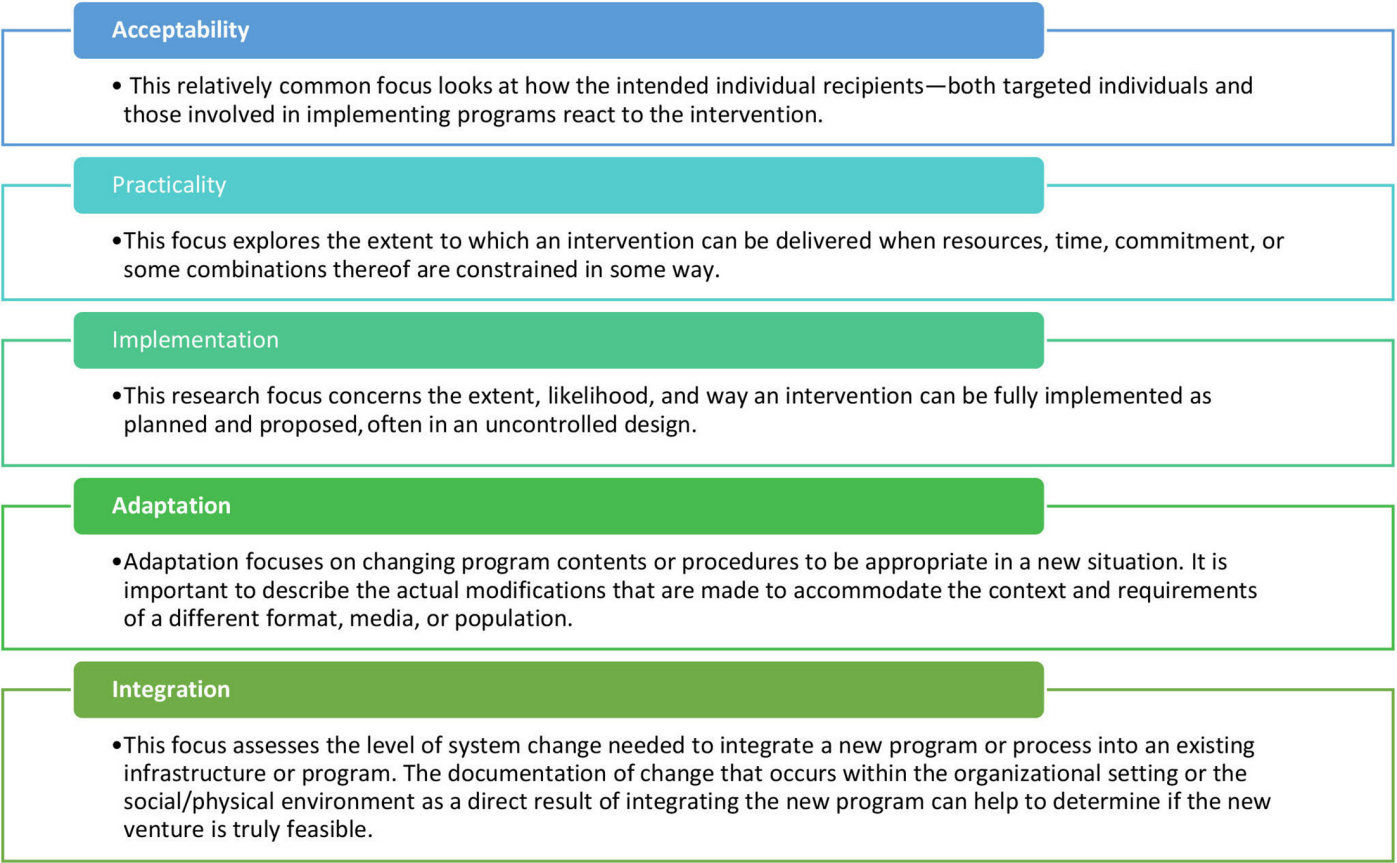
Four domains of feasibility of digital health interventions adopted in this study.

This study used a qualitative descriptive approach to assess the feasibility of digital health in enhancing access to health services. A framework was adapted to gather the perspectives of demand-side and supply-side stakeholders regarding the feasibility of digital health in enhancing healthcare access (refer to figure 1). The adapted feasibility framework has five domains, namely acceptability, practicality, implementation, adaptation and integration. Internet-based mapping was done to identify existing digital health service platforms in Karachi, Pakistan.^29^

### Study setting and participants

This study was conducted as part of a five-country research titled ‘m-Consulting as an option for communities with minimal healthcare access in low-resource settings’.^30^ The study was funded by the UK Research and Innovation, technically supported by the University of Warwick (UK) and was implemented in Pakistan, Tanzania, Kenya, Nigeria and Bangladesh.

**Figure 2:**
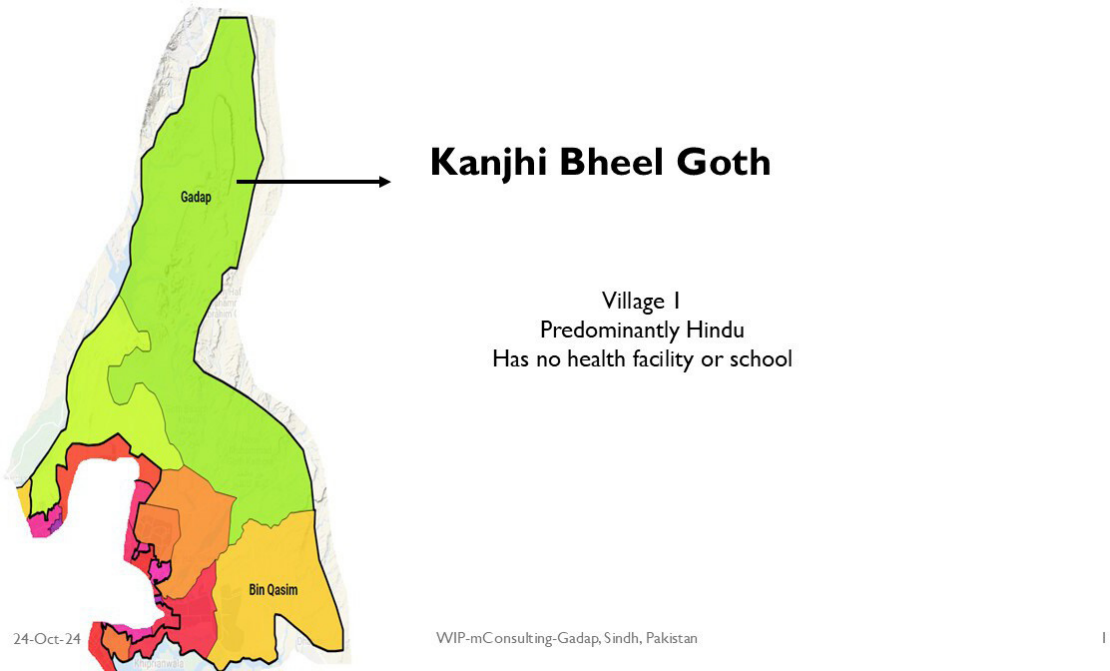
Map of village 1 in Gadap.

In Pakistan, the study was carried out at three villages of Gadap, a periurban area in Karachi. These three villages were included in the study due to their variant socioeconomic contexts and difficulties with access to healthcare. Figures2^4^ show the map of the field sites. The total area of the town of Gadap has an area of 1200 km, with one million population, and has a population density of 91 people/square km. This area has eight union councils, covering >400 villages. Housing is a mix of mud and brick structures.^31^ Most houses have access to electricity/solar panels, and household members have low literacy levels: 38% in men and 12% in women.^32^

**Figure 3:**
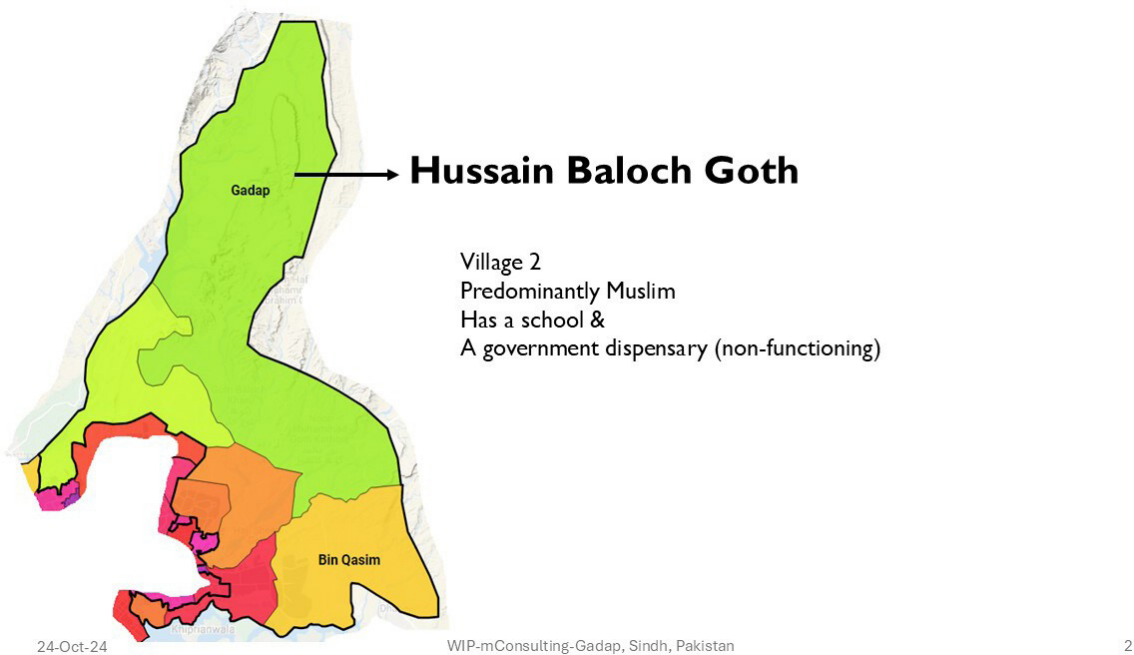
Map of village 2 in Gadap.

**Figure 4:**
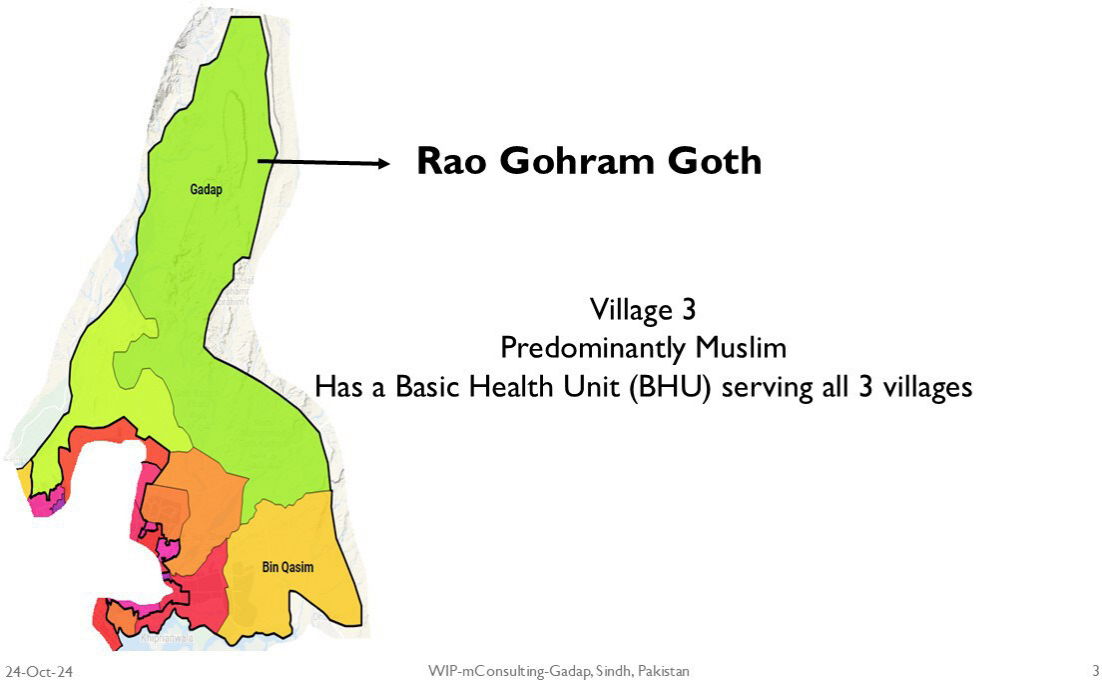
Map of village 3 in Gadap.

The demand-side stakeholders included community leaders such as religious leaders (n=7); community activists, including schoolteachers, social mobilisers and representatives from non-governmental organisations (n=20); and community members (n=58). The supply-side stakeholders included public and private sector healthcare providers (n=5); paramedics, community-based health workers and pharmacists (n=25); and digital health providers (n=3) and experts (n=3).

### Sampling technique and data collection process

The research team consisted of trained qualitative research scientists, along with a moderator, community liaison officers, note keeper and data collectors. All interviews were conducted in the national language (Urdu). All audio recordings were initially transcribed into Urdu and translated into English by trained qualitative team members. About 10% of the transcriptions were reverse-translated into Urdu to ensure the quality and validity of the content. A purposive sampling approach was used to enrol demand-side and supply-side stakeholders. Purposive sampling was used as it helps in selecting participants who have a deep and comprehensive understanding of the phenomena from a specific perspective or within a specific context.^33^ Moreover, purposive sampling^34^ assists in identifying the cases, individuals or communities that bestcomprise a sample in order to facilitate answering the aims and objectives of the research, thus improving the rigour of the study and the trustworthiness of the data and the results.^35^

Using semistructured guides, key informant interviews (KIIs) with digital health experts and providers, as well as indepth interviews (IDIs) with community activists and leaders and healthcare providers and managers, were conducted to collect data. Mini-interviews (MIs) were conducted with community members (online supplemental material). The interview guides were developed in English, translated into Urdu and then back-translated into English to ensure validity. The KII and IDI interview guides were pilot-tested with six participants, while the MI guide was pretested with five participants. The interview guides had open-ended questions and probes to elucidate the perspectives of the demand-side and supply-side stakeholders on the feasibility of digital health in terms of acceptability, practicality, implementation, adaptation and integration. During discussions, care was taken to avoid stifling the dialogue and to allow opportunities for new domains to emerge.

Data were collected iteratively between March 2019 and March 2020. The KII was conducted at participants’ offices, the IDI was conducted at local community centres and the MI was conducted in the community. To establish rapport, the participants were invited in a cordial manner to voluntarily participate in the interview, with full assurances of anonymity, privacy and confidentiality. The participants then gave their informed written consent for participation on a voluntary basis. The duration of KIIs, IDIs and MIs was 60, 40 and 20 min, respectively. KIIs and IDIs were audio-recorded and were pseudo-anonymised and transcribed. The field notes also included participants’ impressions and emerging topics. Data collection tools are included in online supplemental annexure 2.

Data collection continued until theoretical saturation was reached and no new themes appeared. All data were encrypted, stored and processed by applicable data protection regulations. There were no financial rewards for the participants.

### Data analysis and interpretation

Using deductive and inductive approaches, the data were thematically analysed by identifying codes, categories and themes.^36 37^ Five feasibility framework domains were used for digital health interventions analysis. Findings were clarified using the one-sheet-of-paper method,^38^ and codes were assigned to the data collection techniques and gender of the participants (IDI=indepth interview; KII=key informant interview; MI=mini-interview; F=female; M=male) and to the study site (SS=three villages in Gadap).

Transcriptions were thoroughly read to understand the content collected during data collection. Initial coding was done by deductive approach until no new information came across. Two team members coded the data independently using the NVivo V.12 software, and the principal investigator (PI) of the study resolved all discrepancies.

Triangulation was done across data sources (supply-side and demand-side stakeholders) and methods (KI, IDI and MI) for a comprehensive understanding of the feasibility of digital healthcare in enhancing healthcare access. A four-dimensional criterion proposed by Lincoln and Guba was employed to maintain study rigour, which includes credibility, dependability, confirmability and transferability.^39 40^ The results were shared with the community and the advisory group members to discuss the way forward, which is not within the scope of this paper.

### Patient and public involvement

The study proposal was developed through a thorough online literature search and also involved stakeholders. Further, the study tool was piloted with the help of two stakeholders’ (both a healthcare provider and a community member) input on digital health in the area. This piloting was inclusive and comprehensive. None of the healthcare providers (HCPs) and community members was involved in the development of the research protocol and in standard procedure decisions.

## Results

In total, 152 participants engaged in the study. The participants were diverse in age, ranging from 20 to 70 years. 114 (about 75%) were men, while very few were women (n=38). Participants were mostly healthcare providers or teachers. The KIIs were conducted with digital healthcare providers and experts (n=6), the IDIs with community activists (n=20) and healthcare providers (n=30), the MIs with community members (n=58), and consensus workshops with representation from all these supply-side stakeholders (n=15) and demand-side stakeholders (n=23), including community leaders and members. The data collection techniques along with the types of participants are given in table 1.

**Table 1:** Data collection techniques and types of participants

Number	Data collection technique	Types of participants	n
1	Key informant interviews	Digital healthcare providers and experts	6
2	Indepth interviews	Community activists	20
		Healthcare providers	30
3	Mini-interviews	Community members	58
4	Consensus workshops (n=4)	Healthcare managers (n=2)	15
5		Community leaders (n=2)	23
Total			152

The steps of data analysis, including the codes, categories and themes, are presented in table 2. Based on the data gathered from the supply-side and demand-side stakeholders of digital health services (N=152), the findings are presented in the five domains of the feasibility framework, namely acceptability, practicality, implementation, adaptation and integration.

**Table 2:** Description of themes, categories and codes

Number	Themes	Categories	Codes
1	Acceptability of digital health	Facilitator and benefits.	Use of telephones as a potential solution. Reliable method.
		Access to phones and 24/7 internet.	For phones to avail healthcare services. Internet access.
		Health services.	For new symptoms, side effects or laboratory reports.
		Barriers and concerns.	For stigmatised health issues. Concerns about the quality of digital healthcare.
2	Practicality of digital health	Enhance health service delivery.	Good option in case of emergency. Ease in patient care and follow-up or advice.
		User acceptability.	Bridging gaps in healthcare access. User-friendly and easy approach. Commonly used in the community.
3	Adaptation of digital health	Satisfaction in use.	Local societal values and norms. Online mobile services.
		Regulation guidelines and protocols.	Trustworthy for health workers. Need for reliable guidelines.
4	Implementation of digital health	Barrier to scalability of digital health services.	Lack of awareness about digital health services. Charging mechanism.
		Recommendation.	Creation of digital health services. Need for resources.
5	Integration of digital health services	‘Hub & Spoke Model’.	Stewardship and accreditation of digital health services. Public trust/stakeholders. Collaboration and coordination.
		Reinforce digital policy integrations.	Awareness. Training. Health services. Resource allocation.

### Acceptability of digital health services

All participants emphasised that the use of telephones is a potential solution to enhance access to healthcare services, especially for semiurban and rural residents.

Use of telephones for getting advice is a potential solution for our health problem [for accessing healthcare]. (Community MI, Community Member 6)

All categories of participants, including healthcare providers and community members and leaders, reported that the use of mobile phones for healthcare advice is acceptable in their communities. Many community members (21 out of 58) shared their experiences of using mobile phones to avail healthcare services. Community members, who shared their experiences of using mobile phones to avail healthcare services (n=21), reported that they used mobile phones for taking follow-up appointments, seeking clinical advice after working hours or during emergencies, or for informing the healthcare provider about new symptoms, side effects or laboratory reports.

Community members call their doctors to inform them about their lab reports or side effects of drugs to get guidance about medication. (Community MI, Community Members 5 & 10)

Of the community members who reported using their mobile phones to consult with healthcare workers (21 out of 58), most (18 out of 21) did not recognise their practices to be under ‘Digital Health/Tele-Health’/’m-Consulting’*.* Of those who used mobile phones to access healthcare, very few community members (3 out of 58) consulted telehealth clinics using mobile phones to receive individual healthcare advice.

Healthcare providers concurred on the favourable aspects of digital health and validated the use of mobile phones for accessing healthcare from healthcare workers. Community members further identified that the anonymity of this mode makes it an ideal platform for patients to discuss sensitive or stigmatised health issues. Moreover, community members informed that patients prefer face-to-face services from private clinics and pharmacies rather than using digital health platforms. They further reported that community members approach private-for-profit pharmacists either in person or through phone with queries about minor illnesses and emergencies.

Many patients prefer going to the pharmacy and getting medicines rather than calling a person they do not know. (IDI, Community Activist 4)

Pharmacists validated this information and reported receiving calls from community members for healthcare advice.

Patients call us to get treatment for minor illness and in emergencies. We listen to their symptoms, prescribe, and sell medicines, and sometimes make referrals to doctors or hospitals. No consultation fee is charged for these mConsulting, only the cost of drugs is taken. (IDI, Pharmacist 1)

Community members also voiced concerns about the quality of digital healthcare, fearing that it might introduce additional costs and time if they need inperson care. They expressed that it is stressful to discuss health issues with an unknown person. They further mentioned that digital healthcare is not a replacement for inperson physical examination.

In person physical examination consultation is the primary mode of healthcare delivery. How will the healthcare provider do the physical examination that is essential for identifying the main problem and prescribing the right treatment. (Community MI, Community Member 7)

Most healthcare providers (22 out of 30) also valued inperson examination and reported that it is a more reliable method to assess for signs of an illness.

Comparison of face-to-face consultation with digital is impractical. For instance, now, if […] I am consulting with somebody [via mConsulting] now; I cannot check how pale the person is. […] Even if you do a video, if I say let me see your tongue, the way I would see it in a video might be different from when I see it [physically]. (IDI, Healthcare Provider 2)

All participants expressed that digital health could not be a complete replacement for face-to-face care.

### Practicality of digital health

The participants unanimously reported that digital healthcare is a good option in case of emergency. All stakeholders mentioned that digital health is a practical solution to getting health advice from healthcare providers, especially for minor ailments, health problems after working hours, emergency illness and if a healthcare provider is not available in town.

My friend got operated on and something was wrong with his stitches. The doctor was not available in town, so he called him on the phone and the doctor prescribed the medicine. (IDI, Healthcare Provider 3, Basic Health Unit)

Healthcare providers reported that currently they do not call patients or community members for follow-up or advice. However, they confirmed that they mostly receive calls from patients to get advice during emergencies, such as for high-grade fever, diarrhoea or vomiting.

I received a call from one of my patients that his son is vomiting. I gave him emergency advice by telling him to give his son Oral Rehydration Salt solution, I also instructed him to take his son to the nearest health care center. (IDI, Private Healthcare Provider 8)

Community members also reported that digital health is practical as it saves finances and time.

Taking appointment through mobile phone saves time and transport costs. (Community MI, Community Member 25)

Healthcare providers confirmed that when community members acquire any illness, they first seek healthcare advice remotely before visiting the health facility.

If any community member become sick, he/she enquires from us first about the type of drugs that she/he might use in such a condition before they opt for going to the facility for more treatment and spending a lot. (IDI, Healthcare Provider 16)

Healthcare providers emphasised the practical implications of digital health in enhancing healthcare accessibility, especially for women facing mobility constraints due to prevailing gender norms. Digital health experts mentioned that digital health has a crucial practical role in bridging gaps in healthcare access and enhancing access to healthcare.

A few community members (5 out of 58) reported interrupted electric supply and poor internet connectivity as the main practical barriers to digital health. Furthermore, in a majority of households, mobile phones, especially smartphones, were owned by male members. All healthcare providers reported that low technological literacy and lack of formal mechanisms for charging consultation fees are practical barriers to providing digital healthcare.

We advocate for the establishment of user-friendly fee payment mechanisms. (IDI, Healthcare Provider 22)

Some healthcare providers (10 out of 30) expressed their discomfort in using technology. For many, the idea of formally undertaking digital health provokes anxiety and apprehension. They believed a lack of proper training in technology and its use creates fear and apprehension.

I believe that healthcare providers are inadequately trained in technology and its use for delivering health services creates fear and apprehension among them. (KII, Healthcare Manager, Non-Governmental Hospital)

Few healthcare providers (12 out of 30) were concerned that digital health would negatively impact the demand for face-to-face services and, with this, their livelihood.

### Implementation of digital health

All participants were informed that there is lack of awareness about digital health and its platforms among the public.

People lack information about these services [Digital health services]. (Consensus Workshop 1, Participants 1 and 5)

All the demand and supply-side stakeholders reported that before moving towards implementation of digital health, the curcial initial step should be to raise awareness among community members about the significance and the process of accessing healthcare through digital health.

Before implementation of digital health, awareness raising about its benefits and process should be the first crucial step. (KII, Digital Health Expert 2 and Digital Health Provider 1; Consensus Workshop 1, Participants 2 and 8)

Technology experts also concurred and strongly recommended raising awareness among community members about existing digital health platforms.

We strongly recommend increased awareness among community members about existing mConsulting platforms. (KII, Digital Expert 2)

Furthermore, healthcare providers pointed out that the absence of formal digital health consultation charging mechanisms results in potential revenue loss. They highlighted the need to design fee payment mechanisms. Moreover, all participants suggested the creation of digital health services that involve all stakeholders, including technology experts, healthcare providers, patients, and community leaders’ and members.

Digital health services should be co-planned by all stakeholders including technological experts, healthcare providers, patients and community leaders’ community and members. (IDI, Healthcare Providers 9 and 12, KII, Digital Health Expert 1, Consensus Workshop 1, Participants1 and 7)

A system should be developed for facilitating technologically illiterate and less resourceful patients to access digital health services .

### Adaptations of digital health

All stakeholders suggested that a few adaptations are essential for the inclusion of digital health as a mode of healthcare delivery into the existing health service delivery model. Community members suggested that, to tailor digital healthcare services to local societal values and norms, the first healthcare consultation should be face to face, either through inperson visit to the clinic or through online mobile services. This face-to-face consultation is crucial in building trust between patients and healthcare providers, which is fundamental for better healthcare outcomes.

Face-to-face consultation either online or in-person helps in developing trust in healthcare providers. Once patients start trusting their healthcare providers, they follow all their suggestions. (Consensus Workshop 1, Participants 4 and 7)

Healthcare providers suggested that it is crucial to train HCPs on digital techology and its use in healthcare service delivery for enhancing their confidence to use this mode for health services.

We strongly recommend digital technology trainings to boost healthcare providers’ confidence in delivering healthcare through this mobile mode. (IDI, Healthcare Providers 2 and 4)

Digital health experts suggested that digital health implementation regulation guidelines and protocols be developed.

### Integration of digital health services

All stakeholders highlighted that state stewardship and accreditation of digital health services will enhance public trust in this mode of healthcare delivery.

For building people’s trust in the services [digital health services], should be [delivered] under the Government. (Consensus Workshop 1, Community Member 10)

Community members and their leaders and local healthcare providers recommended a healthcare model where grass-root workers could be trained as field coordinators to raise awareness about digital healthcare and serve as a focal person for connecting patients with healthcare providers at secondary-level and tertiary-level facilities.

In this healthcare model, lady health workers/volunteers and community members should train to raise awareness about digital health. The healthcare providers at primary healthcare facilities could connect patients remotely to healthcare providers working at secondary and tertiary facilities. (IDI, Healthcare Provider 4)

Similarly, digital health experts also recommended a ‘Hub & Spoke Model’ to smoothly incorporate digital health into the local healthcare infrastructure, within the framework of a more extensive telemedicine strategy. These experts further suggested that fieldworkers could train in digital technologies to raise awareness about digital healthcare and to improve access to quality healthcare through connecting primary with secondary-level and tertiary-level facilities.

Technology experts suggested that *tele-devices* for basic laboratory testing and *technological apps* for maternal and child health education ought to be incorporated into this ‘Hub and Spoke Model’.

Tele-devices [e.g., for basic laboratory testing] and apps [e.g. for maternal and child healthcare] could be incorporated into this approach too. (KII, Digital Expert 2)

Digital health experts flagged that, despite having a digital health policy, digital health has yet to be fully integrated into the healthcare system.

Although Pakistan has a digital health policy, however lack of clarity about digital healthcare services in details. (KII, Digital Expert 3)

## Discussion

In this study, all the demand-side and supply-side stakeholders reported that the use of mobile phones to seek health advice is acceptable and practically feasible in semiurban and rural communities and emphasised that it is a potential solution to enhance access to healthcare by vulnerable populations. These findings have been endorsed by a mixed-methods study conducted in Bangladesh.^41^ Literature also reassures the potential of digital health in enhancing access to healthcare, often reflecting on the ubiquity of mobile phones and the ever-increasing connectivity globally, reaching remote or otherwise disengaged populations.^42^ Digital healthcare has been highly effective in improving the health of the population in many countries in the world.^43^ Digital healthcare has become more relevant to Pakistan, where the health sector is underfunded^44^ and the health system faces diverse challenges, including inadequacy of healthcare infrastructure and massive shortage of human resources.^30 45 46^ Moreover, Pakistan has significant urban and rural health inequality^47^; 63% of the rural population receives a meagre portion of the health budget. To enhance access to basic service packages, Pakistan’s government needs to explore innovative strategies to improve the efficiency of its resource-constrained health system,^48^ and digital health is one of the most viable options, as proven by studies.^30 49^ The feasibility of digital healthcare is also supported by the fact that 75% of the Pakistani population has mobile phone subscriptions.^50^ There is therefore a dire need to use the potential of digital health in addressing the health needs of the population and enhancing access and equity in healthcare.

**Figure 5:**
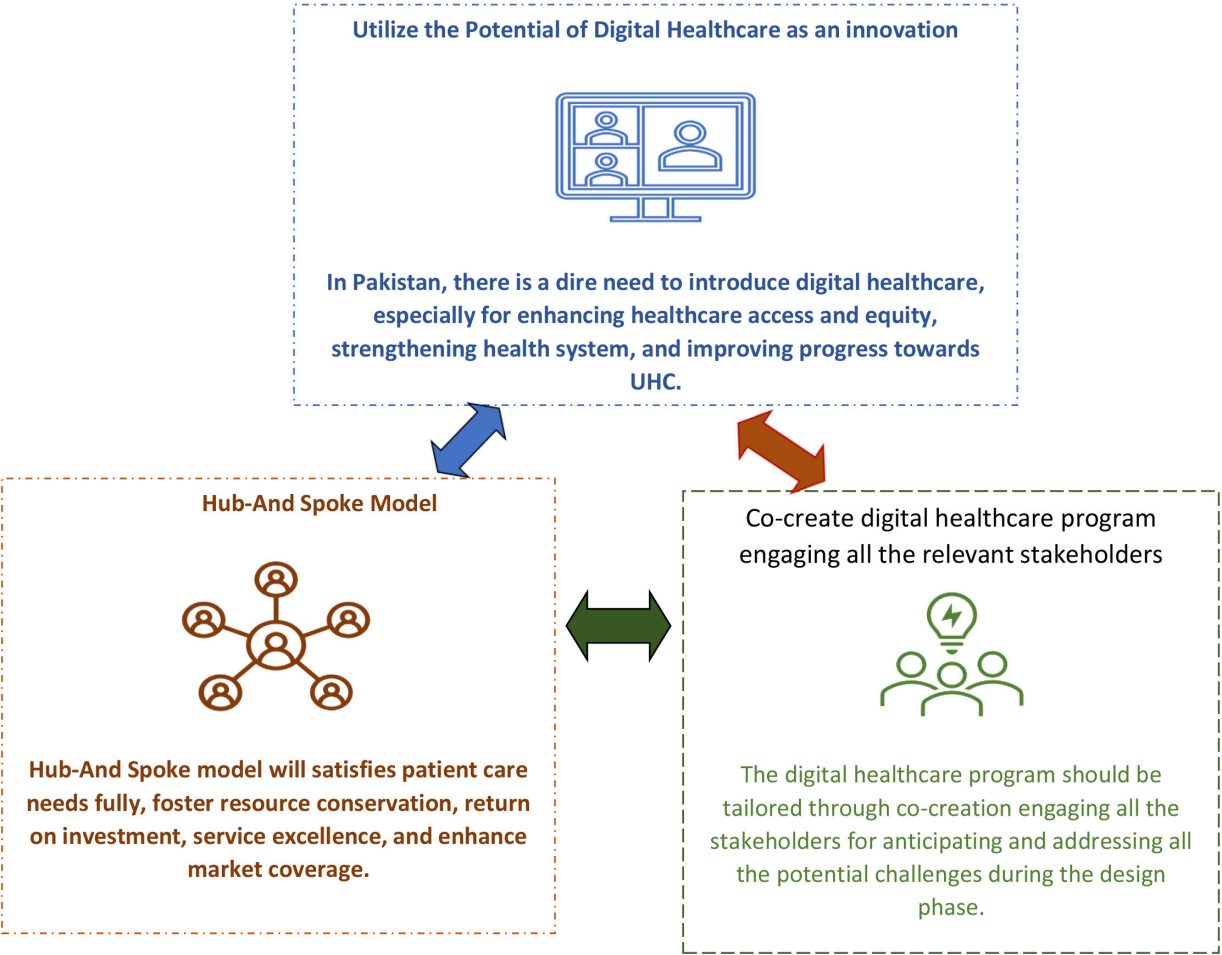
Feasibility of digital health in enhancing access to healthcare in Pakistan. UHC, universal health coverage.

This study has identified that digital health should not completely replace inperson healthcare and suggested a mix of inperson and digital consultation. Literature also rejects complete replacement of inperson healthcare with remote or virtual alternatives.^51^ Evidence also confirms that a balance between inperson and remote healthcare services might be the most effective way to provide comprehensive healthcare, as this approach can ensure that specialised care is still available while expanding access to general healthcare services through remote or satellite locations^52^,^54^ and also improving clinical outcomes.^55^ A good example of a mix of inperson and digital consultation is a ‘Hub-And-Spoke Organization Design’, one of the avenues that offer great potential for serving patients well.^53 56^ The hub and spoke model has origins in the transportation industry but has been adopted by and used successfully in many other industries, including retailing, education and healthcare.^53 57^ The hub and spoke model organisation design is a model that arranges service delivery assets into a network consisting of an anchor establishment (hub), which offers a full array of services, complemented by secondary establishments (spokes), which offer more limited-service arrays, routing patients needing more intensive services to the hub for treatment.^53 58 59^ Through strategic centralisation of the most advanced medical services at a single site and distribution of basic services via secondary sites, the hub and spoke model affords unique opportunities to maximise efficiency and effectiveness.^58^ Literature proves that a well-designed hub and spoke model network satisfies patient care needs fully, yet does so in a manner that fosters resource conservation, return on investment, service excellence and enhanced market coverage.^60^ Based on the findings of this study and the existing robust evidence, it is suggested that LMICs, including Pakistan, should design digital healthcare services using a hub and spoke model (refer to figure 5).

This study identified few challenges to implementing digital health, including privacy, ethical issues, lack of evidence-based standards, weak management systems, effectiveness of digital services, training of healthcare providers, technological barriers and access to digital health services by vulnerable populations, such as the elderly, women, individuals who are illiterate and those of low-income class, due to low access to technology and low health literacy level. Similar implementation challenges have been reported by studies in other countries.^61^,^65^ Participants emphasised that the digital health programme should be tailored according to the norms, values, sociocultural, economic and technological context of Pakistan. Literature also validates that digital healthcare programmes need to be tailored according to the health needs and social norms of LMICs to minimise implementation challenges.^66^ Tailoring healthcare has proven to be an effective and cost-effective method for achieving desired health outcomes.^65^ Tailoring is one process that involves selecting and/or modifying strategies to address contextual factors that potentially influence implementation.^65^ It is considered a critical step to supporting successful delivery of evidence-based interventions in healthcare.^67^ During the tailoring process, potential implementation challenges from the perspectives of all stakeholders should be anticipated and thoroughly addressed through comprehensive planning. In healthcare service systems, stakeholders often have conflicting goals, including quality of life, accessibility, trust, safety, convenience, patient-centredness and communication.^68^ To address these conflicting goals and tailor digital healthcare according to the contextual factors, co-creation can be a viable option for establishing a sustainable partnership across stakeholders.^69^ Co-creation is the participation of users in the development process, and its application significantly improves the quality, usability and social acceptance of new solutions.^70^ Based on the study findings and the existing knowledge, we suggest that digital healthcare should be co-created through stakeholders engagement for anticipating potential challenges and making plan to address those during the implementation.

## Conclusion

This study concludes that digital healthcare is acceptable and practically feasible in semiurban and rural communities and is a potential solution to enhance access to healthcare by vulnerable populations. The study highlights that digital health should not completely replace inperson healthcare, but should be offered as a mix of inperson and digital consultation, the hub and spoke model. Few challenges to implementing digital health exist, including privacy, ethical issues, lack of evidence-based standards, weak management systems, effectiveness of digital services, training of healthcare providers, technological barriers and access to digital health services by vulnerable populations, such as the elderly, women, individuals who are illiterate and those of low-income class, due to low access to technology and low health literacy level. The findings of this study collectively underscore the overwhelmingly positive reception and perceived value of digital health within the healthcare system. These findings imply that digital health possesses the potential to instigate substantial improvements within the healthcare sector. As such, these results provide valuable insights into the feasibility and desirability of integrating digital health as a transformative element in healthcare service delivery, promising a more accessible, efficient and patient-centric healthcare experience.

## Recommendations

Based on stakeholders’ perspectives, our study suggests three main recommendations for a successful integration of digital health within the existing healthcare: use the potential of digital health as an innovation; use the hub and spoke model as a model that is a mix of inperson and telehealth components; and co-create digital healthcare programme engaging all relevant stakeholders to address existing implementation challenges and to successfully implement and sustain this innovation. Further research would be beneficial to comprehensively understand the local and regional healthcare landscape and disparities and tailor digital healthcare as per their unique characteristics.

## Supplementary material

10.1136/bmjopen-2023-082558 online supplemental file 1

10.1136/bmjopen-2023-082558 online supplemental file 2

## Data Availability

Data are available upon reasonable request.
