# Supplementary material for: Feasibility of digital healthcare in enhancing healthcare access in semiurban areas of Karachi, Pakistan: a qualitative descriptive study
Source: BMJ Open. 2025 Jul 11;15(7):e082558. doi: 10.1136/bmjopen-2023-082558 (PMC12273075; doi:10.1136/bmjopen-2023-082558)
Supplement: online supplemental file 1 [file bmjopen-15-7-s001.pdf]

Feasibility of Digital Health for Enhancing Access to Healthcare in semi-urban Karachi, Pakistan: Stakeholders' Perspectives

Guide for Key Informant & In-Depth Interviews

Domain-1: General Information about Mobile Phone, Internet & Use of Mobile Health Services

1.1. Are mobile phone services available in Gadap?

Probes:

- If yes, are these available everywhere & to everyone?
- If not, where are these available & to whom?

1.2. Do people have access to a mobile phone?

Probes: If yes, probe to find out:

- How many have mobile phones? (Ask how many out of 10)
- Ask separately for men and women
- Does your mobile phone work locally?
- Which provider do you use?

1.3.What is the current situation with regard to internet availability?

Probes: Ask in relation to:

- Infrastructure
- Coverage
- Users
- Issues & challenges

1.4. Do people have access to the internet?

Probes: If so, probe to find out:

- How many have access to the internet? (Ask how many out of 10)
- Ask separately for men and women (Ask how many out of 10)
- Is internet connection available 24/7?

- If not, how many hours it is available?

Domain-2: Acceptability- This relatively common focus looks at how the intended individual recipients—both targeted individuals and those involved in implementing programs —react to the intervention.

2.1. Do people use mobile phones to get advice or help about their health? (Community health worker, pharmacist, nurse, doctor or traditional healer)?

Probes:

- If yes, ask:
  - What do they do?
  - Who did they contact?
  - Which services do they use?
  - What benefits do these mobile health services provide?

- If not, why not.

2.2. Have you ever used your mobile phone to get advice or help with someone else's health?

Probes:

- If yes, ask:
  - What do you do?
  - Who did you contact?
  - Which services do you use?
  - What benefits do these mobile health services provide?

2.3. Do you know anyone else who has used a mobile phone to get advice about their or someone else's health?

Probes: If yes, could you explain with the help of example:

- What do you do?
- Who did you contact?
- Which services do you use?
- What benefits do these mobile health services provide?

2.4. Are there any formal mobile health services available for people living in Gadap Town to discuss a health need with a healthcare provider (Community health worker, pharmacist, nurse, doctor or traditional healer) by using their mobile devices (e.g. phones, tablets)?

Probes: If yes, probe about:

- Type of services
- For how long
- Size
- Purpose/Aim of these services
- Coverage
- Operating system
- Cost

2.5.If a mobile health service is initiated for people living in Gadap Town to discuss a health need with a healthcare provider (Community health worker, pharmacist, nurse, doctor or traditional healer) by using their mobile devices (e.g. phones, tablets), would people use those?

Probes: Ask:

- If yes, why?
- If not, why not?

Domain-3: Practicality- This focus explores the extent to which an intervention can be delivered when resources, time, commitment, or some combinations thereof are constrained in some way.

3.1. What would be the response of different demand and supply side stakeholders towards mobile health services in your area?

Probes: From the perspective of:

- Clients/Patients
- Healthcare providers
- Community members
- Community leaders
- Religious leaders

3.2. What could be the possible challenges for initiating mobile health services in your area?

Probes: In terms of:

- Access to mobile phone
- Availability of internet
- Women's health issues
- Cultural issues
- Economic perspective
- Community in terms of acceptability, values, norms, traditions

3.3.What could be the possible strategies for addressing these challenges for initiating mobile health services in your area?

Probes: How could the above-mentioned challenges be addressed in following areas:

- Access to mobile phone
- Availability of internet
- Women's health issues
- Cultural issues
- Economic perspective
- Community in terms of acceptability, values, norms, traditions

3.4. Your perceptions about the benefits of mobile use for healthcare in terms of:

Probes:

- Patients/Clients
- Healthcare provider
- Health services specially availability, access, quality
- Family specially cost
- Community in terms of acceptability, values, norms, traditions

3.5. Your perceptions about the harms of mobile use for healthcare in terms of:

Probes:

- Patients/Clients
- Healthcare provider
- Health services specially availability, access, quality
- Family specially cost
- Community in terms of acceptability, values, norms, traditions

Domain-4: Implementation- This research focus concerns the extent, likelihood, and manner in which an intervention can be fully implemented as planned and proposed,6 often in an uncontrolled design.

4.1.In your opinion, could mobile health services be initiated in your area?

Probes: To find out:

- If yes, why?
- If not, why not?

4.2. What should be the scope and package of mobile health services at the start?

Probes: To find out their perspective in relation to:

- Maternal
- Child
- Infections
- Emergency
- Non-Communicable
- Any other

4.3. What steps should be taken for implementing mobile health services in your area?

Probes: To find out who should be involved and how:

- Patients
- Community members
- Leaders-Political, religious
- Healthcare providers
- Any other

4.4. What would the effect/impact of 'mobile health services' on healthcare delivery system.

Probes:

- Effect/impact on other healthcare provision
- Healthcare seeking & access
- Healthcare quality

Domain-5: Adaptation- Adaptation focuses on changing program contents or procedures to be appropriate in a new situation. It is important to describe the actual modifications that are made to accommodate the context and requirements of a different format, media, or population.[7]

5.1. In your opinion, what modes and mechanisms should be used for delivering mobile health services in your area?

Probes: To find out:

- Individual services
- Facility-based
- Community Health Workers/Lady health workers as medium
- Any other.

5.2.What modifications need to be made in the healthcare delivery system for integration of mobile health services in your area?

Probes: To find out:

- Behaviors
- Infrastructure
- Technology
- Training
- Resources
- Any other

5.3.What innovations need to be made in the healthcare delivery system for integration of mobile health services in your area?

Probes: In the following areas:

- Behaviors
- Infrastructure
- Technology
- Training
- Resources
- Any other

Domain-6: Integration- This focus assesses the level of system change needed to integrate a new program or process into an existing infrastructure or program.[8] The documentation of change that occurs within the organizational setting or the social/physical environment as a direct result of integrating the new program can help to determine if the new venture is truly feasible.

6.1. What changes will be required in the existing healthcare delivery system for integration of mobile health services?

Probe to find out:

- Infrastructure
- Fee structure
- Payment mechanism
- Health workforces
- Health services

6.2. Who are the key stakeholders in this area who should be involved during the planning of integration of mobile healthcare services into the existing healthcare delivery system?

Probes: To find out who should be involved and how:

- Patients
- Community members
- Leaders-Political, religious
- Healthcare providers
- Any other

6.3. Which strategies can be used to bring about these changes so that mobile health services can be integrated into the health service delivery model?

Probe to find out:

- Use of social media platforms
- Use of Community-Based workers
- Training of healthcare providers
- Behavior Change Communication
- Advocacy
- Any other
