# Supplementary material for: Feasibility of digital healthcare in enhancing healthcare access in semiurban areas of Karachi, Pakistan: a qualitative descriptive study
Source: BMJ Open. 2025 Jul 11;15(7):e082558. doi: 10.1136/bmjopen-2023-082558 (PMC12273075; doi:10.1136/bmjopen-2023-082558)
Supplement: online supplemental file 2 [file bmjopen-15-7-s002.pdf]

Annexure I: Operational Definitions

- **'Hub & Spoke Model' (HSM):** The creative architecture of *'Hub & Spoke Model'* (HSM**)** places a strong emphasis on making the best use of the limited healthcare resources available in rural locations. HSM shows that even in the most remote locations, medical care can be using contemporary techniques and tools at a far lower final cost. Through the application **of** the HSM, poor peripheral settings can be strengthened by linking them to a hub with an abundance of resources. Such a model may fit right in with the resource-poor health systems that are typical in low- and middle-income countries. A peripheral plant can receive material support from the resource-rich hub as well as its technical know-how and skilled workforce[71, 72].
- **Digital Health:** We defined digital health as "when a person, with a perceived health need, consults a healthcare provider using mobile communication technology, or a provider contacts patient." We categorized digital health services into two main types:
- **Digital Health Platforms:** These platforms are run by commercial companies, government agencies or non-governmental organizations (NGOs). Health services provide by using written communication (text messaging, app-based information, and web chats) audio and/or video channels. Consultants include real people and algorithm-driven computers.
- **Individual Digital Health Services:** This is a personal phone-based health service undertaken by either individual community members to consult their healthcare provider or local healthcare workers to speak to community members. Health workers include pharmacists, community health workers, nurses, clinical officers, and doctors.
