# Supplementary material for: Feasibility of digital healthcare in enhancing healthcare access in semiurban areas of Karachi, Pakistan: a qualitative descriptive study
Source: BMJ Open. 2025 Jul 11;15(7):e082558. doi: 10.1136/bmjopen-2023-082558 (PMC12273075; doi:10.1136/bmjopen-2023-082558)
Supplement: Supplementary file 3 [file bmjopen-2023-082558.reviewer_comments.pdf]

PEER REVIEW HISTORY

BMJ Open publishes all reviews undertaken for accepted manuscripts. Reviewers are asked to complete a checklist review form and are provided with free text boxes to elaborate on their assessment. These free text comments are reproduced below.

ARTICLE DETAILS

Title (Provisional)

Feasibility of Digital Healthcare for Enhancing Healthcare Access in Semi-Urban Karachi, Pakistan: A Qualitative Descriptive Study

Authors

Rizvi, Narjis; Iqbal, Romaina; Jabeen, Rawshan; Harris, Bronwyn; Griffiths, Frances

---

VERSION 1 - REVIEW

**Reviewer**	**1**
**Name**	**Pannarunothai, Supasit**
**Affiliation**	**Centre for Health Equity Monitoring Foundation**
**Date**	**16-Dec-2023**
**COI**	**No competing interests**

The paper was drafted based on a multi-centred study covering many countries, taking only Pakistan for the present format.

To increase to quality of the paper, I would suggest:

1. Describe the hub and spoke model clearly as it is the main model recommended based on the study. Was it suggested by the informants or by the researchers? A lot of research should be further studied if the directions of developments take this path.

2. More characteristics of key informants should be presented. Age would be the most interesting attribute concerning adoption or experiences in exposure to digital health platform. The paper presents somewhere that male got hold of smart phones more than female, then gender attribute might be interesting. The counting of different categories of respondents should be cautious, such as line 181, ...very few community members (total n=6/152) were aware of... should it be 6/58?

3. The uses of terms should be clearer, such as line 205 ... they did not recognize... who are they? Or whether because of terms 'digital health', 'tele-health' are confusing then 'they' got confused. Line 452 ...three main strategies ..., it is difficult to pinpoint at the conclusion what are the three.

4. Referring to who said that quote should not display Pakistan as the paper selects only Pakistan.

5. Annex should list on the semi-structured questions, as it curious how the hub and spoke arose.

6. Typo error, line 452 ... prospective ... should be ...perspective.

**Reviewer**	**2**
**Name**	**Nkangu, Miriam**
**Affiliation**	**University of Ottawa**
**Date**	**01-Apr-2024**
**COI**	**None**

Paper reports on the feasibility of implementing digital health from a stakeholder perspective, the paper could be strengthened with the following suggested points

Background

Lines 71-73 should be revised, “Realizing digital health as a potential solution for enhancing access to healthcare, many digital health initiatives had been launched but did not progress beyond the research phase and were not implemented” this is an over statement, and it is not clear what the authors are referring, there are digital health platforms that have been scaled, but most did not reach scaled due to limited funding, government buy-in etc. Authors should be clear what they mean by beyond the research phase, digital health has various stages from, prototype, development/pilot testing, scale, and evidence generation.

Lines 73-75, authors should provide examples of the digital platform that uptake is slower or failed to be sustained to substantiate this sentence.

The authors should reorganize the background in a chronological and structured approach. The paragraph on digital health should be introduced before telemedicine, because telemedicine is a form of digital health, currently the authors describe telemedicine, then digital health and back to telemedicine, it is not clear if authors are looking at the feasibility of telemedicine or the feasibility of digital health with a focus on telemedicine.

Authors should provide in the background what they mean by demand(patient/?) and supply-side(health facility, providers?? factors within the health system they are focusing their study.

Authors should provide reference to this statement to substantiate this given that it is the statement that is defining the problem for the study and provide examples of such digital platforms to strengthen this sentence “One of the main causes of failure in becoming a sustainable service model is the insufficient planning to ascertain stakeholders needs and

technological requirements before the implementation of 80 a digital healthcare service model.”

Authors should state in a sentence or two, if there are any existing digital platforms in the context they are focusing the study, what has been done, and how this study will help inform the existing gap, considering that the feasibility exploration was focused in a specific context, there is no mention of any digital health tools or platforms that have been implemented in that context before this study and/or the nature of the digital landscape to strengthen the background. Are authors planning to introduce a digital platform and want to scope the context and feasibility??

The sentence about ethical review board is incomplete, it should be well defined, the name of the ethics board and location. If more than one ethics was obtained, it should be stated.

Data collection: who conducted the interviews and FGD, in what language? how were the tools defined and assessed? Pretesting? where was the interview conducted? And FGD, how many participants were involved in the FGD, how were participants selected for the FGD or who was eligible? How did authors achieve or defined saturation? How was the interview conducted? What kind of questions were being explored?

Data analysis is not well explained, how many people did the coding? What software was used or how was the coding planned? how were deductive and inductive codes derived? For each themes? describe the process of the coding. How was agreement obtained? How was the quality of the analysis assured? Did they conduct member checking?

What do the authors mean by this sentence “The findings were translated into English while preserving their culturally specific meanings”.?

Are the authors referring to transcription being translated into English before coding? if so, they need to provide additional information in the data collection section about the instrument if it was designed in English before being translated to the local language and vice versa?

Can the authors expand on what they mean by this sentence “After the completion of the analyses, the summary reports were disseminated and the results were then reviewed, discussed, and finalized”?

This sentence requires additional information, triangulation of what??? And how was the triangulation done? “Triangulation was done from a supply and demand side perspective to have a comprehensive understanding of the feasibility of mConsulting in enhancing healthcare access”.

Line 146 to 147 Is not clear, were results shared for feedback or shared as part of dissemination.? Depending on what the objective was for sharing the results will determine if this should go under results section or data analysis. lines 148 to 157 should be moved to results.

Results

Authors have not described the outcome of the coding, the first paragraph should describe study participants, the second paragraph should list the themes that constituted the results derived from the data analysis process and possible categories, before proceeding to describe the themes in detail.

This paper requires a substantial amount of revision and reorganize the structure of the paper. the results section starts with the review of the digital landscape, however, the objective points to this as a secondary aspect. This section should actually go into the background and the authors should keep the objective simple in line with the qualitative interviews and FGD on the feasibility. The internet mapping etc. is part of the literature review that authors should conduct to build the background of the paper and therefore should constitute part of the background to support the objective. Currently, there is no background detail about digital health in the context of the study or country, lines 160-182 should be moved to the background and reorganize the flow.

In the results section, authors should revise the word informed as used in a couple of sentences which should either be refer to as participants

---

VERSION 1 - AUTHOR RESPONSE

Reviewer's Comments	Authors Response
Reviewer: 1<br>1. To increase to quality of the paper, I would suggest:<br>1.1. Describe the hub and spoke model clearly as it is the main model recommended based on the study.<br>1.2. Was it suggested by the informants or by the researchers?<br>1.3. A lot of research should be further studied if the directions of developments take this path.<br>2. More characteristics of key informants should be presented.<br>2.1. Age would be the most interesting attribute concerning adoption or experiences in exposure to digital health platforms. The paper presents somewhere that male got hold of smart phones more than female, then gender attribute might be interesting.	1. For quality improvement:<br>1.1. 'Hub and Spoke' model is clearly explained (Line 586=594, Page-25 & 25)<br>1.2. It was suggested by both- the informants and the researchers (Line 404 - 406, Page-7)<br>1.3. The need for further research is added in the way conclusion and recommendation. (Line 559, Page 22)<br>2. Characteristics of key informants

2.2. The counting of different categories of respondents should be cautious, such as line 181, ...very few community members (total n=6/152) were aware of... should it be 6/58?<br><br>3.<br>3.1. The uses of terms should be clearer, such as line 205 ... they did not recognize... who are they?<br>3.2. Or whether because of terms 'digital health', 'tele-health' are confusing then 'they' got confused.<br><br>3.3. Line 452 ...three main strategies ..., it is difficult to pinpoint at the conclusion what are the three.<br><br>4. Referring to who said that quote should not display Pakistan as the paper selects only Pakistan.<br><br>5. Annex should list semi-structured questions, as it curious how the hub and spoke arose.<br><br>6. Typo error, line 452 ... prospective ... should be ...perspective.	2.1. Age and gender characteristics of participants is added. (Line 203-205, Page 9)<br><br>2.2. Corrected across results section. (Line 264, Page 12; Line 272, Page 13; Line 273, Page 13; Line 301, Page 14; Line 341, Page 15; Line 348, Page 15; Line 355, Page 15.)<br><br>3.<br>3.1. Text rephrased to make it clear (Lines 264-265, Page 12).<br>3.2. Text rephrased to explain that all those who used mobile phones for availing healthcare services did not relate it to ‘Digital health’/’Tele-Health’/’m-Consulting’. (Lines 274, Page 13)<br><br>3.3. Three recommendations are added in the text in the section on ‘Conclusions & Recommendations (Lines 559-564, Page 22)<br><br>4. Pakistan is removed from all the quotes in the result section (Line 260-Page 12; Lines 271,287 & 293- Page 13; Lines 302 & 326-Page 14; Lines 331, 337, 349 & 356-Page 15; Lines 362, 368, 372 & 381-Page 16; Lines 394, 400 & 419-Page 17; Lines 424 & 429, Page 18.)

	5. Semi-Structured Guide for FGD & Key Informant Interviews is added as Annex<br><br>6. Typo corrected (Line 559, Page 22)
**Reviewer: 2**<br>**1. Background**<br>1.1 Lines 71-73 should be revised, “Realizing digital health as a potential solution for enhancing access to healthcare, many digital health initiatives had been launched but did not progress beyond the research phase and were not implemented” this is an over statement, and it is not clear what the authors are referring, there are digital health platforms that have been scaled, but most did not reach scaled due to limited funding, government buy-in etc. Authors should be clear what they mean by beyond the research phase, digital health has various stages from, prototype, development/pilot testing, scale, and evidence generation.<br>1.2. Lines 73-75, authors should provide examples of the digital platform that uptake is slower or failed to be sustained to substantiate this sentence.<br><br>1.3. The authors should reorganize the background in a chronological and structured approach. The paragraph on digital health should be introduced before telemedicine, because telemedicine is a form of digital health, currently the authors describe telemedicine, then digital health and back to telemedicine, it is not clear if authors are looking at the feasibility of telemedicine or the feasibility of digital health with a focus on telemedicine.	1.1. Text revised (Lines76-84, Page 4)<br><br>1.2. References are added to substantiate this sentence and presenting the examples of the digital platform where either the uptake was slow or the project could not be sustained (Line 84, Page 4)<br><br>1.3. Background is reorganized in a chronological and structured approach (Lines 57-61, Page 3)

1.4. Authors should provide in the background what they mean by demand(patient/?) and supply-side (health facility, providers?? factors within the health system they are focusing their study.<br>1.5. Authors should provide reference to this statement to substantiate this given that it is the statement that is defining the problem for the study and provide examples of such digital platforms to strengthen this sentence "One of the main causes of failure in becoming a sustainable service model is the insufficient planning to ascertain stakeholders needs and technological requirements before the implementation of 80 a digital healthcare service model."<br><br>1.6. Authors should state in a sentence or two, if there are any existing digital platforms in the context they are focusing the study, what has been done, and how this study will help inform the existing gap, considering that the feasibility exploration was focused in a specific context, there is no mention of any digital health tools or platforms that have been implemented in that context before this study and/or the nature of the digital landscape to strengthen the background. Are authors pl Page 1anning to introduce a digital platform and want to scope the context and feasibility?<br>1.7. The sentence about ethical review board is incomplete, it should be well defined, the name of the ethics board and location. If more than one ethics was obtained, it should be stated.<br>1.8. Data collection: who conducted the interviews and FGD, in what language? how were the tools defined and assessed? Pretesting? where was the interview conducted? And FGD, how many participants were involved in the FGD, how were participants selected for the FGD or who was eligible? How did authors achieve or defined saturation? How was the interview conducted? What kind of questions were being explored?	1.4. The background of demand and supply-side factors is added (Lines 95-101, Page 4)<br><br>1.5. The reasons for the failures of implementation of digital health models is added and references are provided. (Lines 76-81, Page 4)<br><br>1.6. A paragraph is added about the existing digital situation in the context where the study was conducted. (Lines 85-94, Page 4)<br>There have been plans to introduce a digital platform and therefore the scope is to understand the context and feasibility.<br><br>1.7. The full name of the ethical review board is

1.9. Data analysis is not well explained, how many people did the coding? What software was used or how was the coding planned? how were deductive and inductive codes derived? For each theme? describe the process of the coding. How was agreement obtained? How was the quality of the analysis assured? Did they conduct member checking?<br><br>1.10. What do the authors mean by this sentence "The findings were translated into English while preserving their culturally specific meanings".?<br><br>1.11. Are the authors referring to transcription being translated into English before coding? if so, they need to provide additional information in the data collection section about the instrument if it was designed in English before being translated to the local language and vice versa?<br>1.12. Can the authors expand on what they mean by this sentence "After the completion of the analyses, the summary reports were disseminated and the results were then reviewed, discussed, and finalized"?<br>1.13. This sentence requires additional information, triangulation of what??? And how was the triangulation done? "Triangulation was done from a supply and demand side perspective to have a comprehensive understanding of the feasibility of mConsulting in enhancing healthcare access".<br>1.14. Line 146 to 147 Is not clear, were results shared for feedback or shared as part of dissemination.? Depending on what the objective was for sharing the results will determine if this should go under results section or data analysis. lines 148 to 157 should be moved to results.<br>2. Results	added. (Lines 13-14 Page 1, 117-118, Page 5)<br><br>1.8. All the details about data collection are added including: who conducted the interviews, in what language? how were the tools defined and assessed? Pretesting? where was the interview conducted? how participants were selected or who was eligible? How did authors achieve or defined saturation? How was the interview conducted? What kind of questions were being explored? (Lines 145-164 Pages 6 & 7)<br>The tool is also added as annex 1.<br>1.9 All the details about data analysis is added including: how many people did the coding? What software was used or how was the coding planned? how were deductive and inductive codes derived? How was agreement obtained? How was the quality of the analysis assured? Did they

2.1. Authors have not described the outcome of the coding, the first paragraph should describe study participants, the second paragraph should list the themes that constituted the results derived from the data analysis process and possible categories, before proceeding to describe the themes in detail.<br><br>2.2. This paper requires a substantial amount of revision and reorganize the structure of the paper. the results section starts with the review of the digital landscape, however, the objective points to this as a secondary aspect. This section should actually go into the background and the authors should keep the objective simple in line with the qualitative interviews and FGD on the feasibility. The internet mapping etc. is part of the literature review that authors should conduct to build the background of the paper and therefore should constitute part of the background to support the objective. Currently, there is no background detail about digital health in the context of the study or country, lines 160-182 should be moved to the background and reorganize the flow.	conduct member checking? (Lines 168-191 Pages 7 & 8)<br>1.10. Corrected as-All the audio recordings were transcribed in Urdu and were translated into English by the trained team and 10% of the translations were back translated into Urdu to ensure the quality and validity of the content (Lines 131-132 Page 6)<br>1.11. The data collection semi-structured guide was developed in English, translated in English and then back translated to Urdu. (Lines 149-150 Page 6)<br><br>1.12. Text removed.<br><br>1.13. Triangulation is explained and details added (Lines 188-190 Page 8)<br><br>1.14. Text removed

	2.1. Changes have been made as suggested by review-2: the first paragraph is describing the study participants, the second paragraph is giving codes, categories and themes. (Lines 207-256190 Page 10-11). Table-2 is added describing the data analysis process including codes, categories and themes. (page-11)<br>2.2. The result section is reorganized and the section on the review of the digital landscape has been moved to the background. (Lines 85-94 Page 4)<br>The results section starts with the findings on the feasibility under the major domains. (Lines 260-433 Pages 12-18)

VERSION 2 - REVIEW

**Reviewer**	**1**
**Name**	**Pannarunothai, Supasit**
**Affiliation**	**Centre for Health Equity Monitoring Foundation**
**Date**	**19-Aug-2024**
**COI**	**No competing interest declared**

1. In the title, abstract, strengths and limitations, and conclusion; there are conflicts in defining the areas that the present study aims for.

2. In the Methods, Study settings..., the authors should add more information on 3 villages of Gadap, were they semi-urban (far distance from each other), and how results can recommend for rural, remote rural areas.

3. The hub-and-spoke model explanations/discussions are still unclear. It was imposed by the research team rather than coming from key informants.

VERSION 2 - AUTHOR RESPONSE

Comments	Authors response
Reviewer: 1<br>Dr. Supasit Pannarunothai, Centre for Health Equity Monitoring Foundation<br>Comments to the Author:<br><br>1. In the title, abstract, strengths and limitations, and conclusion; there are conflicts in defining the areas that the present study aims for.<br>2. In the Methods, Study settings..., the authors should add more information on 3 villages of Gadap, were they semi-urban (far distance from each other), and how results can recommend for rural, remote rural areas.<br><br>3. The hub-and-spoke model explanations/discussions are still unclear. It was imposed by the research team rather than coming from key informants.	1. Edits have been made line# 33<br><br>2. Text added 136-147<br><br>3. Digital health experts suggested 'hub-and-spoke model' for enhancing access to quality healthcare for semi-urban and rural residents through training field workers in digital technology.
Reviewer: 1<br>If you have selected 'Yes' above, please provide details of any competing interests.: No competing interest declared	Response 'No' is selected
